# Cell Cycle Dynamics during Stomatal Development: Window of MUTE Action and Ramification of Its Loss-of-Function on an Uncommitted Precursor

**DOI:** 10.1093/pcp/pcad002

**Published:** 2023-01-04

**Authors:** Daniel T Zuch, Arvid Herrmann, Eun-Deok Kim, Keiko U Torii

**Affiliations:** Department of Molecular Biosciences, The University of Texas at Austin, 2506 Speedway, Austin, TX 78712, USA; Department of Molecular Biosciences, The University of Texas at Austin, 2506 Speedway, Austin, TX 78712, USA; Department of Molecular Biosciences, The University of Texas at Austin, 2506 Speedway, Austin, TX 78712, USA; Howard Hughes Medical Institute, The University of Texas at Austin, 2506 Speedway, Austin, TX 78712, USA; Department of Molecular Biosciences, The University of Texas at Austin, 2506 Speedway, Austin, TX 78712, USA; Howard Hughes Medical Institute, The University of Texas at Austin, 2506 Speedway, Austin, TX 78712, USA

**Keywords:** *Arabidopsis*, Cell cycle, Cell division, Live imaging, MUTE, Stomatal development

## Abstract

Plants develop in the absence of cell migration. As such, cell division and differentiation need to be coordinated for functional tissue formation. Cellular valves on the plant epidermis, stomata, are generated through a stereotypical sequence of cell division and differentiation events. In *Arabidopsis*, three master regulatory transcription factors, SPEECHLESS (SPCH), MUTE and FAMA, sequentially drive initiation, proliferation and differentiation of stomata. Among them, MUTE switches the cell cycle mode from proliferative asymmetric division to terminal symmetric division and orchestrates the execution of the single symmetric division event. However, it remains unclear to what extent MUTE regulates the expression of cell cycle genes through the symmetric division and whether MUTE accumulation itself is gated by the cell cycle. Here, we show that MUTE directly upregulates the expression of cell cycle components throughout the terminal cell cycle phases of a stomatal precursor, not only core cell cycle engines but also check-point regulators. Time-lapse live imaging using the multicolor Plant Cell Cycle Indicator revealed that MUTE accumulates up to the early G2 phase, whereas its successor and direct target, FAMA, accumulate at late G2 through terminal mitosis. In the absence of *MUTE*, meristemoids fail to differentiate and their G1 phase elongates as they reiterate asymmetric divisions. Together, our work provides the framework of cell cycle and master regulatory transcription factors to coordinate a single symmetric cell division and suggests a mechanism for the eventual cell cycle arrest of an uncommitted stem-cell-like precursor at the G1 phase.

## Introduction

Multicellular organisms grow and develop through a series of cell divisions, which coincide with the deployment of gene regulatory networks to create diverse, organized tissues. For plants, in which cells are immotile, cellular divisions and growth must be carefully coordinated in order to properly distribute specific cell types and drive tissue shape formation. As such, plants have evolved to utilize multiple environmental and developmental pathways to regulate the cell cycle (Harashima and Schnittger 2010, Shimotohno et al. 2021). Importantly, accumulating evidence has shown that specific cell cycle regimes are closely integrated with the differentiation of specific cell types—allowing plants to dynamically tune cell-type composition, growth and patterning during tissue formation (Inze and De Veylder 2006, Shimotohno et al. 2021).

Core cell cycle engines are highly conserved across eukaryotes and can be divided into four distinct phases [Gap 1 (G1), DNA synthesis (S), Gap 2 (G2) and mitosis (M)] (Johnson and Walker 1999, Harashima et al. 2013). Each cell cycle phase relies upon phase-specific cyclins, cyclin-dependent kinases (CDKs) and transcription factors to drive the cell cycle forward. Temporally regulated synthesis and stability of cyclins/CDKs, as well as CDK inhibitors, tune the activity of cyclin/CDK complexes to ensure the progression and timing of cell cycle phases (Johnson and Walker 1999, Harashima et al. 2013). It has been shown that plants encode a large number of core cell cycle genes and their modifiers, likely reflecting a diverse array of cell cycle states and context-specific modification of cell cycle dynamics (Riou-Khamlichi et al. 2000, Peres et al. 2007, Sozzani et al. 2010, Shimotohno et al. 2021).

Of particular importance to the fitness of the land plants are the development and distribution of stomata, or adjustable pores, on the leaf surface—which facilitate gas exchange and transpiration. Studies in *Arabidopsis* have shown that stomatal differentiation occurs through a series of stereotypical cell divisions controlled by three master-regulatory basic helix–loop–helix (bHLH) transcription factors, SPEECHLESS (SPCH), MUTE and FAMA (Lau and Bergmann 2012, Han and Torii 2016). SPCH initiates and maintains asymmetric cell divisions (ACDs) of stem-like stomatal precursors called meristemoids (MacAlister et al. 2007, Pillitteri et al. 2007). After a few rounds of ACDs, MUTE drives the differentiation of a meristemoid into a guard mother cell (GMC) and simultaneously orchestrates a single, terminal symmetric cell division (SCD) (Pillitteri et al. 2007, Han et al. 2018). Lastly, FAMA terminates the cell cycle in each daughter cell and completes the terminal differentiation of guard cells (Ohashi-Ito and Bergmann 2006).

**Fig. 1:**
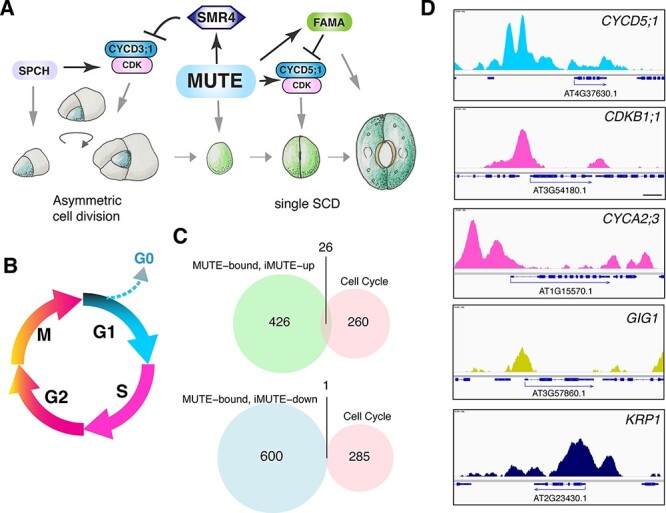
MUTE directly upregulates a suite of cell cycle regulatory genes. (A) Schematic model of how MUTE promotes GMC SCD and stomatal differentiation via cell cycle regulatory genes. (B) Phases of the cell cycle are color-coded according to the PlaCCI fluorophore expression window (phases not to scale). After mitosis, there is a brief period without any fluorescence signal. We defined this stage as ‘early G1’. Withdrawal from the cell cycle (hence the loss of fluorescence) is indicated as G0. (C) Venn diagrams of MUTE bound and *iMUTE*-up (top left), MUTE bound and *iMUTE*-down (bottom left) and a curated cell cycle gene (right). For gene lists, see **Supplementary Dataset S1**. (D) Genome browser view of the ChIP-seq profile of MUTE binding to the promoter of *CYCD5;1, CDKB1;1, CYCA2;3, GIG1* and *KRP1*. Peaks are color-coded according to their general expression window as in (B); *KRP1* is highlighted as navy blue, showing that it is repressed by MUTE.

How the stomatal-lineage bHLH proteins integrate with cell cycle machinery to switch cell cycle modes is an important question, and previous studies have revealed several key links (**Fig. 1A**) (Han and Torii 2019). For instance, SPCH initiates ACDs by inducing the expression of the G1-specific D-Type Cyclin CYCD3;2 (Lau et al. 2014), which is likely complex with CDKA;1 to launch the cell cycle and drive mitotic divisions (Healy et al. 2001, Dewitte et al. 2007). MUTE then disengages the SPCH-mediated ACDs by directly upregulating SIAMESE-RELATED4 (SMR4), which inhibits CYCD3s but permits MUTE-induced G1 cyclin CYCD5;1 to proceed to a terminal SCD (Han et al. 2022). An additional G1 cyclin CYCD7;1 may also coordinate the SCD (Weimer et al. 2018). Lastly, FAMA and the Myb protein FOUR LIPS, which are directly induced by MUTE, suppress the cell cycle by directly inhibiting the expression of multiple core cell cycle genes including *CDKB1;1*, thereby ensuring that the terminal SCD occurs only once (Xie et al. 2010, Hachez et al. 2011). Additional studies have highlighted the importance of the mitotic cell cycle vs. endoreduplication in the epidermal cell–fate decision to stay on the stomatal cell lineage or differentiate into pavement cells (Meyer et al. 2017, Ho et al. 2021).

While recent efforts have uncovered several mechanisms by which the stomatal differentiation programs utilize cell cycle machinery, it remains unclear whether the cell cycle gates the expression of master regulatory stomatal-lineage bHLH proteins. This is most critical for MUTE and FAMA, in which sequential actions occur within a single round of the cell cycle during the terminal SCD. Here, we show that MUTE directly upregulates a suite of cell cycle components through the cell cycle phases during the terminal SCD, whereas both MUTE and FAMA exhibit temporally restricted expression profiles that coincide with specific cell cycle phases. Furthermore, in the absence of *MUTE*, the G1 phase of dividing meristemoids becomes progressively extended as the meristemoids reiterate ACDs. Together, our work highlights the cell cycle windows during which MUTE and FAMA accumulate to coordinate a single SCD and suggests a mechanism for the eventual cell cycle arrest of uncommitted *mute* meristemoids at the G1 phase.

## Results

### MUTE directly induces a suite of cell cycle regulators throughout the cell cycle duration

Previous studies have revealed that SPCH, MUTE and FAMA tightly regulate stomatal development through the control of both differentiation and cell cycle (**Fig. 1A**). Among them, MUTE must precisely coordinate the differentiation and single SCD of meristemoids into pairs of guard cells (Han et al. 2018, 2022). Here, we explore the relationship between MUTE and cell cycle machinery to understand how MUTE can reliably orchestrate the shift from proliferation to a single terminal division. Notably, *MUTE* overexpression (*iMUTE*) was shown to upregulate a suite of genes involved in the cell cycle, cell division and mitosis (Han et al. 2018), together representing a complete and independent cell cycle module (**Fig. 1B**). To address which of those genes are direct MUTE targets, we compared *iMUTE* RNA sequencing (RNA-seq) (Han et al. 2018) and MUTE ChIP sequencing (ChIP-seq) (Han et al. 2022) data to extract a subset of cell cycle genes that are directly up- or downregulated by MUTE (**Fig. 1C**). Among the 286 combined and curated cell cycle genes, 26 are both bound by MUTE and upregulated by *iMUTE* (**Fig. 1C**, **Supplementary Dataset S1**). Those direct MUTE targets include SMR4, a known cell cycle inhibitor that slows down the fast proliferative ACD of a meristemoid, as well as *CYCD5;1*, a known G1 cyclin that subsequently drives the GMC SCD (Han et al. 2018, 2022).

From our analysis, we found that MUTE-direct targets include genes implicated in G1/S check-point control, *E2FF/DEL3 (DP/E2F-like3)* (*AT3G01330*) and *E2FC* (*AT1G47870*), a known component of the DREAM complex (Lang et al. 2021), as well as E2F target gene 1 (*ETG1)* (*AT2G40550*) (**Fig. 1C** and **Supplementary Dataset S1**). In addition, MUTE directly upregulates the expression of the DREAM complex components *TESMIN-LIKE CXCs* (*TCXs*), including *TCX2/SOL2* (*AT4G14770*), *TCX3/SOL1* (*AT3G22760*) and *TCX4* (*AT3G04850*). *TCX2/SOL2* has been identified as stem cell ‘ubiquitous’ genes that likely play a role in cell divisions in diverse plant stem cell populations (Clark et al. 2019). *TCX2/SOL2* and *TCX3/SOL1* are direct SPCH targets, and their loss-of-function mutations confer aberrant stomatal-lineage divisions and occasional misregulation of guard cell fate (Simmons et al. 2019). Our finding that they are also direct transcriptional targets of MUTE is consistent with the roles of *TCX2/SOL2* and *TCX3/SOL1* in the stomatal fate commitment process.

MUTE also directly induces genes attributed to S-phase progression, including the DNA replication gene *MINICHROMOSOME MAINTENANCE 3* (*MCM3*: AT5G46280) (Stevens et al. 2002), *TSO2* (*AT3G27060*) (Wang and Liu 2006) and A-type Cyclin (*CYCA3;2: AT1G47210*) that is highly expressed in the G1-to-S phase (Menges et al. 2005, Takahashi et al. 2010). In addition to G1, G1/S and S-phase genes, G2-associated mitotic cyclin *CYCB2;3* (*AT1G20610*) and A-type Cyclins (*CYCA1;1: AT1G44110* and *CYCA2;3: AT1G15570*), as well as CDKs *CDKB1;1* (*AT3G54180*) and *CDKB2;1* (*AT1G76540*) (Menges et al. 2005, Romeiro Motta et al. 2022), are also identified as direct MUTE targets upregulated by MUTE (**Fig. 1C, D** and **Supplementary Dataset S1**). Furthermore, a gene regulating mitotic progression at the metaphase–anaphase check-point by negatively regulating the anaphase-promoting complex (APC/C), *GIGAS CELL1* (*GIG1: AT3G57860*) (Iwata et al. 2011), is directly bound and upregulated by MUTE (**Fig. 1C, D** and **Supplementary Dataset S1**). In accordance with our findings that MUTE induces machinery that largely favors cell cycle progression, we found that the CDK inhibitor gene, *KIP-RELATE PROTEIN1* (*KRP1/ICK1*; *AT2G23430*), is the sole cell cycle gene directly bound and downregulated by MUTE (**Fig. 1C, D** and **Supplementary Dataset S1**).

### Dynamics of MUTE accumulation during the single terminal division event

**Fig. 2:**
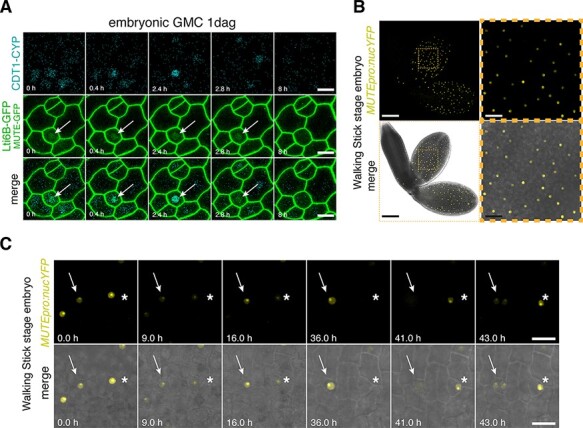
Preformed embryonic GMCs express *MUTE* and execute a terminal SCD upon germination. (A) Representative time-lapse images of preformed embryonic GMCs from 1-day-old cotyledons of germinating WT seedlings expressing *MUTEpro::MUTE-GFP* (nucleus, middle row), cell cycle marker PlaCCI and cell membrane marker Lti6B-GFP. Note that MUTE-GFP starts to accumulate during the G1 phase (CDT1a-CFP, arrow). Scale bars = 10 μm. (B) Expression of *MUTEpro:nucYFP* in a representative walking stick stage WT embryo under a confocal microscope (top) and merged image with brightfield (bottom). Note YFP accumulation within the nucleus in both developing cotyledons. Scale bars = 100 μm. The dotted area is magnified on the right. Scale bars = 20 μm. (C) Time-lapse analyses of representative isolated and cultured walking stick stage embryos expressing *MUTEpro:nucYFP*. One cell expressing *MUTEpro:nucYFP* (embryonic GMC, arrow) undergoes SCD and differentiates into a stoma. Some embryonic cells lose *MUTE* expression after being cultured in a media and initiate de novo ACD (asterisk). Scale bars = 10 μm.

**Fig. 3:**
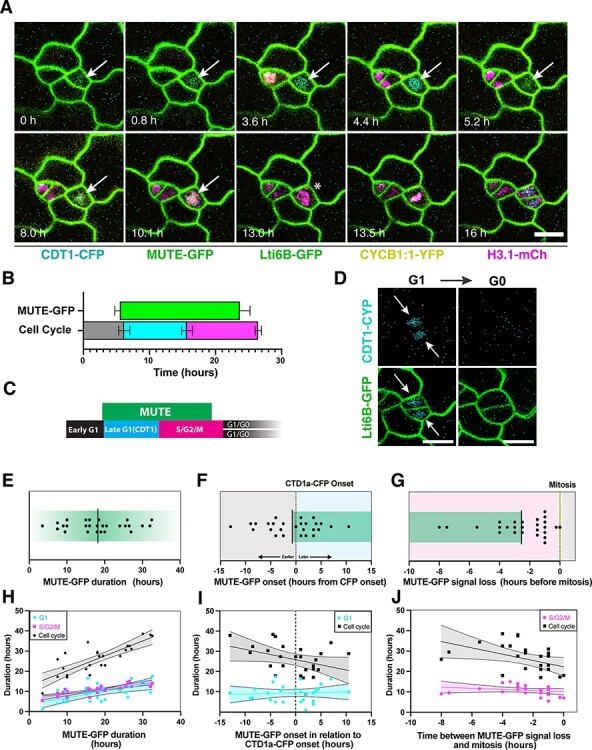
MUTE-GFP expression and cell cycle phase correlation during the terminal stomatal SCD. (A) Representative time-lapse images of a symmetric GMC division of 3-day-old Col-0 cotyledon expressing MUTE-GFP, cell cycle marker PlaCCI and cell membrane marker Lti6B-GFP. Note that MUTE-GFP expression turns on during the G1 phase (CDT1a-CFP, arrow) and vanishes (asterisk) during the S/G2 phase (H3.1-mCherry) prior to mitosis. (B–I) Duration and timing of MUTE-GFP expression and PlaCCI cell cycle phases during stomatal SCDs from time-course confocal images of 3–4-days after germination (dag) Col-0 cotyledons. (B) Measured MUTE-GFP and PlaCCI cell cycle phases. (C) Schematic of expression windows from (B). (D) Representative images of nascent guard cells expressing CDT1a-CFP after division and then losing CDT1a-CFP expression several hours later. (E) MUTE-GFP duration. (F) Time between CDT1a-CFP appearance and MUTE-GFP appearance. (G) Time between MUTE-GFP signal loss and mitosis. (H) Correlation between MUTE-GFP window duration and cell cycle length. (I) Correlation between values in (E) and cell cycle length. (J) Correlation between values in (F) and cell cycle length. Solid lines represent linear regression, and dashed (curved) lines represent the 95% confidence level of linear regression. *n* = 27 from three samples for all. *P*-values: 0.05 > * > 0.005, 0.005 > ** > 0.0005, 0.0005 > *** > 0.00005, 0.00005 > ****. Values and correlation statistics are listed in **Supplementary Dataset S2**.

Because our survey of MUTE-direct targets identified cell cycle genes throughout the cell cycle (**Fig. 1** and **Supplementary Dataset S1**), we sought to address the dynamics of MUTE protein accumulation in the specific context of the single terminal division of a GMC. It has been shown that functional MUTE-GFP proteins become detectable shortly after the last ACD of amplifying meristemoids (Han et al. 2018). To simultaneously monitor the dynamics of MUTE protein accumulation and cell cycle phase, we generated transgenic *Arabidopsis* plants expressing both *MUTEpro::MUTE-GFP* and the multicolor Plant Cell Cycle Indicator (PlaCCI) (Desvoyes et al. 2020) (**Figs. 2A**, **3A****–****C**, **Supplementary Figs. S1, S2 and Videos S1, S2**). PlaCCI is composed of three fluorescent-protein-tagged markers, CDT1a-eCPF, which marks the G1 phase; HTR13-mCherry, which is notable in the S-G2-M phase, and N-CYCB1;1-YFP, which marks mitotic events (Desvoyes et al. 2020) (see **Fig. 1B** and **Supplementary Fig. S1**).

Our long-term time-lapse live cell imaging of germinating cotyledon epidermis revealed two distinct types of GMCs. The first type does not exhibit any sign of asymmetric amplifying divisions and immediately proceeds to the GMC division upon germination (**Fig. 2A** and **Supplementary Video S3**). To address if these GMCs were generated during embryogenesis, we examined the *MUTE* transcriptional reporter (*MUTEpro::nucYFP*) expression during embryogenesis. The walking stick stage embryos from fully expanded mature siliques express discrete spotted patterns of *MUTEpro::nucYFP* in the cotyledons (**Fig. 2B**). To investigate whether these embryonic cells assume future GMCs upon germination, we next performed time-lapse live cell imaging of isolated walking stick stage embryos (see the Materials and Methods section). Indeed, each of the *MUTE-*expressing cells in isolated, cultured embryos directly underwent a terminal SCD and formed a stoma (**Fig. 2C** and **Supplementary Video S4**). We thereby classified them as preformed GMCs during embryogenesis. These cells were omitted from further analysis.

The second type exhibits a typical amplifying ACD, indicative of their initial identity as meristemoid mother cells, expresses *MUTE* following their last ACD and then executes a terminal, single SCD of GMCs to form a pair of guard cells (**Fig. 3** and **Supplementary Videos S1, S2, S5**). We therefore categorized these cells as those that underwent post-embryonic *de novo* progression of stomatal-lineage development (‘post-embryonic *de novo* GMCs’).

For these cells fully undergoing stomatal-cell-state transitions, MUTE-GFP accumulation became evident shortly (4.8 ± 3.6 h) after the last ACD, 0.7 ± 5.4 h prior to CDT1-eCFP (cyan) appearance (**Fig. 3A****–****C, E, F**). The MUTE-GFP signals persisted through G1, S and G2 phases, lasting an average of 18.2 ± 7.9 h (**Fig. 3A****–****C, E**, **Supplementary Fig. S2**), and then disappeared an average of 2.5 ± 2.0 h before the M phase marked by N-CYCB1;1-YFP (yellow) (**Fig. 3A****–****C, G** and **Supplementary Fig. S2**). Following the terminal SCD mitosis, the G1 phase is reinitiated in daughter nascent guard cells, apparent from a brief window of CDT1a-CFP expression (G1), after which the cells appear to exit the cell cycle (G0) (**Fig. 3D**). The duration and onset time of MUTE-GFP correlate well with cell cycle length (**Fig. 3H****–****J**), suggesting that MUTE and cell cycle regulators are co-regulated. At the same time, a larger gap between the disappearance of MUTE-GFP and the subsequent mitosis correlates with a longer overall cell cycle, but not with S/G2/M length, highlighting the likelihood that MUTE onset occurs at a relatively fixed point in G1 and then sustains for a fixed period that is not determined by G2 (**Fig. 3J**). Taken together, our analysis revealed the presence of two classifications of GMCs, embryonic and post-embryonic, both undergoing the single terminal division. More importantly, the simultaneous time-lapse imaging of MUTE and PlaCCI revealed that MUTE accumulation initiates immediately after the last ACD of a meristemoid, sustains through G1-S-G2 and disappears prior to the M phase, consistent with the finding that MUTE transcriptionally induces cell cycle genes, which function through each of the cell cycle phases (**Fig. 1**).

### FAMA turns on in the late G2 phase before the terminal division

**Fig. 4:**
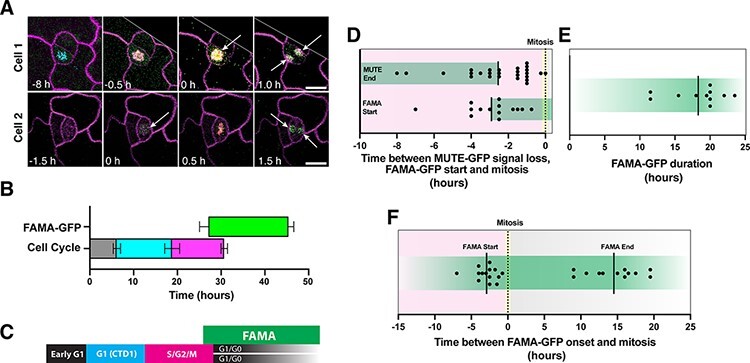
FAMA-GFP expression during the terminal stomatal SCD. (A) Representative time-lapse confocal imaging of 3-day-old cotyledon expressing FAMA-GFP, cell cycle marker PlaCCI and cell membrane marker PM-RB (Plasma Membrane Red Basta). Arrows indicate FAMA-GFP expression before and after mitosis. Scale bars, 10 µm (B–F) Duration and timing of FAMA-GFP expression and PlaCCI cell cycle phases during stomatal SCDs from time-course confocal images of 3–4-days after germination Col-0 cotyledons. (B) Measured FAMA-GFP and PlaCCI cell cycle phases. (C) Schematic of expression windows from (B). (D) Time between FAMA-GFP appearance and mitosis compared to the time between MUTE-GFP disappearance and mitosis [from (F)]. (E) Duration of FAMA-GFP expression. (F) Time between FAMA-GFP appearance and mitosis (left); time between FAMA-GFP disappearance and mitosis (right). *n* = 14 from three samples.

It has been shown that MUTE directly induces *FAMA* expression. This in turn creates a regulatory network motif that can generate a single pulse of cyclin/CDK expression during the terminal division (Han et al. 2018). To examine in which cell cycle phase FAMA accumulates, we generated transgenic *Arabidopsis* plants expressing both *FAMApro::FAMA-GFP* and the cell cycle indicator PlaCCI (**Fig. 4A** and **Supplementary Fig. S3**). Time-lapse imaging analyses of post-embryonic GMCs show that FAMA-GFP accumulation begins in the late G2 phase, an average of 2.9 ± 1.6 h prior to the terminal GMC division (**Fig. 4A****–****D** and **Supplementary Fig. S3**), closely overlapping with the end of the MUTE-GFP expression window (**Fig. 4D**). This is consistent with the previous inducible *MUTE* study, which showed delayed induction of FAMA at 8 h after the induced *MUTE* overexpression (Han et al. 2018). The duration of FAMA-GFP accumulation is nearly identical to that of MUTE-GFP, lasting 18.3 ± 3.9 h on average (**Fig. 4D**). FAMA-GFP persists into G0 of the sister guard cells, terminating 14.5 ± 3.7 h after mitosis (**Fig. 4F**).

### Meristemoids that cannot commit to differentiation extend G1 as reiterating ACDs

**Fig. 5:**
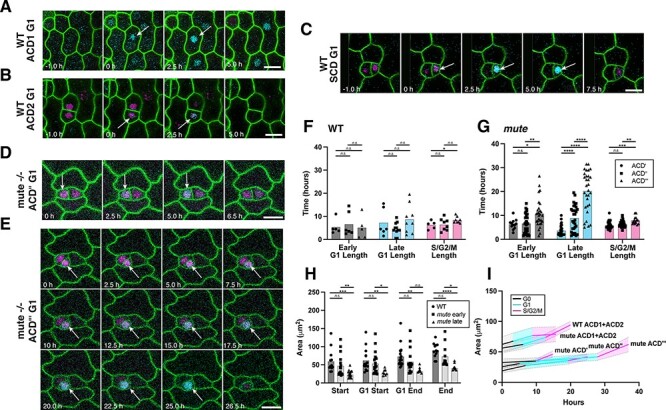
G1 phase is extended in reiterative ACDs in the absence of MUTE. (A–E) Representative time-lapse confocal images of the G1 phase of ACD1 (A), ACD2 (B) and SCD (C) in WT, and ACD″ (D), ACD‴ (E) in *mute* mutants from 1- to 5-day-old cotyledons expressing both PlaCCI and Lti6B (green). ACD1, first ACD; ACD2, second asymmetric amplifying division. ACD″-ACD‴ are the continuous sequence of successive ACDs from the same meristemoid imaged. Arrows indicate the nuclei with CDT1-CFP fluorescence, representing G1. Scale bar, 10 μm. (F and G) Comparison of individual cell cycle length of three rounds of ACD between WT (F) and *mute* mutants (G). ACD′-ACD″-ACD‴ are the continuous sequence of successive ACDs from the same meristemoid imaged. For WT, ACD1, *n* = 7; ACD2, *n* = 11, ACD3, *n* = 13 and samples = 6. For *mute,* ACD′, *n* = 30; ACD″, *n* = 30; ACD‴, *n* = 30 and samples = 5. (H) Area of control and *mute* meristemoids at various stages of the cell cycle. *mute* early refers to initial 1–2 divisions in 2-days after germination (dag) cotyledons; *mute* late refers to ACDs in 3–4-dag cotyledons. For each cell, the area was measured just after a new cell wall formed (start), at CTD1a-CFP onset (G1 start), at CTD1a-CFP disappearance (G1 end) and just prior to cytokinesis (end). Control *n* = 20, *mute n* = 18. WT samples = 6, *mute* samples = 5. (I) Growth through each phase of the cell cycle using individual area values from (H) and average values for phase length from representative divisions. WT ACD1 + ACD2, *n* = 8, *mute* ACD1 + ACD2, *n* = 10, *mute* ACD′ *n* =4, ACD″, *n* = 4, *mute* ACD‴, *n* = 8. ACD′-ACD″-ACD‴ is the continuous sequence of successive ACDs from the same meristemoid imaged. WT samples = 6, *mute* samples* = *5. Scale bars = 10 μm. For pairwise comparisons, a two-tailed Student’s *t*-test was used. *P*-values: 0.05> * > 0.005, 0.005 > ** > 0.0005, 0.0005 > *** > 0.00005, 0.00005 > ****. Values and correlation statistics are listed in **Supplementary Dataset S2**.

In the *Arabidopsis*-developing cotyledon and leaf epidermis, meristemoids typically undergo approximately three proliferative ACDs prior to terminally differentiating into a GMC under the control of MUTE (Nadeau and Sack 2002, Han and Torii 2016). In the absence of *MUTE*, the meristemoids continue to asymmetrically divide, reduce in size and eventually arrest (Pillitteri et al. 2007). To understand the cell cycle behaviors of these *mute* meristemoids that are incapable of differentiation, we quantitatively analyzed the cell cycle dynamics of each round of ACDs in wild-type (WT) and *mute* meristemoids using time-lapse imaging (**Fig. 5**, **Supplementary Figs. S4–S8 and Videos S5, S6**). No significant differences in the early or late G1 duration were observed when comparing sequential divisions in control plants, and a mean difference of only 2 h was observed in the S/G2/M duration between meristemoids undergoing division one compared to division three (**Fig. 5A, B** and **Supplementary Figs. S4–S8**). On average, early G1 (mitosis to CDT1a-CFP onset) lasted for 8.5 ± 4.3 h, late G1 lasted for 6.6 ± 5.0 h and the remaining three phases together (S, G2 and M) lasted for 7.2 ± 2.2 h, for a total cell cycle of 13.8 h starting at CDT1a-CFP onset, consistent with the previous finding (Han et al. 2022), and the entire division cycle for 22.3 h (**Fig. 5F**, **Supplementary Fig. S5 and Video S5**). Also, consistent with the previous findings (Han et al. 2022), CDT1a-CFP expression in GMCs prior to the symmetric terminal division lasted about 5 h (**Fig. 5C** and **Supplementary Fig. S6 and Videos S1, S2**).

Interestingly, we observed an elongation of the ACD cell cycle over successive divisions in *mute* meristemoids, but not in the WT meristemoids. The lengthening of each phase of the cell cycle with successive meristemoid divisions is accompanied by a significant extension of the G1 phase, in particular late G1. As shown in **Fig. 5D, E, G** (see also **Supplementary Figs. S7, S8 and Video S6**), the average late G1-phase duration was increased by roughly fivefold, from 3.65 ± 2.2 h in the first division to 20.18 ± 8.4 h in the third division. Next, to ascertain if G1, specifically, is being lengthened to allow small *mute* meristemoids to expand to a required division threshold, we measured the cellular area of the meristemoids from WT and *mute* mutants throughout the cell cycle in each round of ACDs. There is no significant difference in the meristemoid size between WT and *mute* during ACD1 and 2 in the cotyledon, but that *mute* meristemoids during ACD3 and onward are overall smaller (**Fig. 5H**). We further found that cellular growth of meristemoids is not restricted to G1 but is in fact relatively uniform (**Fig. 5D, E, H, I**). While G1-phase extension may be driving the cellular growth of meristemoids for further ACDs, it is also possible that the gradual G1-phase extension during ACDs of *mute* meristemoids likely reflects a stepwise progression toward developmental arrest, a phenotype that has been observed in aged, uncommitted meristemoids (Pillitteri et al. 2008).

## Discussion

In this study, we investigated the role of MUTE in governing the cell cycle during the terminal division cycle of stomata and whether MUTE expression itself is reciprocally gated by the cell cycle. Furthermore, we revealed that the loss of *MUTE* has phase-specific impacts on cell cycle dynamics in meristemoids, which are not able to switch from a proliferative mode to differentiation. From comparative MUTE ChIP-seq and induced *MUTE* RNA-seq analyses, we have shown that direct MUTE targets extend from G1 (e.g. *CYCD5;1*) (Han et al. 2018) to the G1/S check-point, S-phase and G2 to G2/M check-point, indicating that MUTE is capable of installing a module of cell cycle machinery that is sufficient to execute a complete division cycle. The installation of a complete MUTE-derived cell cycle module has important implications—it may provide opportunities for transcriptional regulatory input by incorporating specific cell cycle components into the developmental network logic. Particularly, the direct upregulation of check-point control components by MUTE, including G1/S transcription factors E2FF/DEL3 and E2F2, S-phase DNA helicase MCM3 and M-phase APC/C regulator GIG1, likely ensures that SCD is executed once *MUTE* is expressed above a certain threshold. In this regard, it is worth mentioning that ectopic *MUTE* overexpression could induce SCD in epidermal cells with a pavement cell–like character, cells that would otherwise be division incompetent (Pillitteri et al. 2007, 2008, Han et al. 2018).

Using the cell cycle marker PlaCCI in conjunction with MUTE-GFP, we found that MUTE-GFP signals always overlap with the G1 and S/G2 phases and are confined to a single division cycle (**Fig. 3**). This restricted accumulation window may reflect feedback from cell cycle machinery or simply be a function of protein stability and non–cell cycle network logic. However, our data also show that MUTE duration correlates with cell cycle length (**Fig. 3H**) and that MUTE-GFP accumulation always diminishes prior to mitosis (**Fig. 3G**, **Supplementary Fig. S2**), suggesting the possibility that MUTE protein is regulated, at least in part, by cell cycle machinery. To date, no studies have identified promoter elements or protein motifs in *MUTE* that resemble known cell cycle regulatory mechanisms: previous analyses on the *MUTE* promoter identified L1 boxes and putative DNA-binding with one finger motifs, neither likely provides direct cell cycle regulation (Peterson et al. 2013, Mahoney et al. 2016). A peak of H3K27me3 at the *MUTE* locus (Lee et al. 2019) could be an opportunity for cell cycle–dependent RETINOBLASTOMA-RELATED1-based chromatin remodeling. Future studies to manipulate the cell cycle lengths and address the effects on MUTE accumulation duration may inform whether *MUTE* expression itself is under cell cycle control.

We found that FAMA, a known target of MUTE, begins to accumulate several hours prior to the SCD at the late G2 phase, coinciding with the timing of MUTE protein disappearance (**Fig. 4D**). The rapid disappearance of MUTE concurrent to FAMA appearance implies a possible mutually exclusive nature of regulation. Indeed, MUTE-induced cell cycle machinery, including *CYCD5;1* and *CDKB1;1*, persists in the GMC in the absence of *FAMA*, triggering multinumeral divisions in *fama* GMCs (Ohashi-Ito and Bergmann 2006, Han et al. 2018). This *fama* phenotype is consistent with the notion that MUTE remains active in the absence of *FAMA*. While the actual, molecular mechanism of this mutual exclusiveness is unknown, it likely involves an epigenetic mechanism. Indeed, as the seedling ages, the *MUTE* locus becomes epigenetically silenced with the deposition of repressive chromatin marks, H3K27me3, which may limit the developmental windows of *MUTE* expression (Lee et al. 2014). FAMA can act as an epigenetic regulator of stomatal-lineage genes, including *SPCH* (Lee et al. 2019). Alternatively, sequential expression of *MUTE* and *FAMA* may reflect their expression windows within the cell cycle, mimicking a MUTE–FAMA mutual exclusion relationship through opposing cell cycle inputs during G2. It would be an interesting future direction to investigate the intersection of G2-specific transcriptional regulation (e.g. MYBR3s) (Kobayashi et al. 2015) on the expression of *MUTE* and *FAMA*.

Previous studies have shown that the cell cycle within the stomatal cell lineage is pliable (Han et al. 2022). Depending on the presence or concentration of CKIs such as the MUTE-induced *SMR4*, the decelerated cell cycle can impact cellular differentiation and fate segregation.

We found that, as meristemoids continue to divide in the absence of *MUTE*, their G1 phase becomes extended in successive divisions (**Fig. 5G, I**). This is different from the MUTE-orchestrated deceleration of the cell cycle in SCD, given that different sets of core cell cycle regulators are operating (Han et al. 2022). Cell cycle arrest in *mute* meristemoids could be a consequence of cell size being a limiting factor to support mitotic division. Based on such a hypothesis, the observed specific G1-phase extension implies that the G1 period might be utilized for cellular growth, our observations that growth occurs in a relatively uniform manner throughout the cell cycle (**Fig. 5H, I**), as well as those of other studies in mammals (Zetterberg and Larsson 1985, He et al. 2009), suggest that G1 may simply be the most pliable/dynamic phase and G1 extension may represent a strategy to increase the cell cycle duration, and thus growth, overall.

We propose that, in meristemoids, the G1 phase operates as a flexible GO-NO-GO threshold, after which the other phases of the cell cycle proceed at a nearly constant rate, until mitosis. Since MUTE protein accumulation starts in G1 (**Fig. 3**), it is also conceivable that G1 lengthening in the absence of MUTE is a survival strategy—providing a larger activation window for MUTE in aging meristemoids. In contrast to the fate-mixing and trans-fating observed due to the early stomatal cell lineage expression of *SMR4*-induced G1 elongation (Han et al. 2022), *mute* meristemoids retain proliferative status presumably due to extended action of the MUTE’s predecessor *SPCH*, which promotes mitotic potential of the meristemoids (Lau et al. 2014). This suggests that cell cycle alteration alone is not sufficient to cause fate-mixing of stomatal guard cells and non-stomatal pavement cells in the absence of *MUTE*. Future studies of how cell cycle machinery shapes the expression of *MUTE* and other stomatal master regulatory transcription factors will illuminate our understanding of specialized cell type differentiation in plants.

## Materials and Methods

### Plant materials

*Arabidopsis* accession Columbia (Col-0) was used as the WT. The following mutants/transgenic lines have been published elsewhere: *mute-2*, used as the *mute* mutant (Pillitteri et al. 2008); *MUTEpro::MUTE-GFP* (Pillitteri et al. 2007); *MUTEpro::nucYFP* (Qi et al. 2017); *FAMApro::FAMA-GFP* (Han et al. 2018); Lti6b-GFP (Kurup et al. 2005) and PlaCCI (Desvoyes et al. 2020). Higher-order mutant/marker lines were generated by genetic crosses, and their genotypes were confirmed. Sterilized *Arabidopsis* seeds were grown on half strength of Murashige and Skoog (MS) media with 1% sucrose and stratified for 2–3 d at 4°C. The seedlings were grown at 22°C under long-day conditions, and 10- to 14-day-old seedlings grown on MS media were transplanted to soil to harvest seeds. For the selection of *mute-2/+*, seeds were grown on 1/2 MS media supplemented with 50 μm/l kanamycin (Fisher, BP906-5).

### Confocal microscopy and time-lapse imaging

Confocal microscopy images were acquired using either Stellaris-8 FALCON (Leica - Mannheim, Germany) using a 63× oil lens for high-resolution imaging and 40× oil lens for data acquisition or SP5-WLL/Argon (Leica) using a 63× water lens for high-resolution imaging and 20× dry lens for data acquisition. The time-lapse imaging of germinating cotyledons expressing PlaCCI (Desvoyes et al. 2020) in WT and *mute*, or together with MUTE-GFP and FAMA-GFP, was performed as described previously (Peterson and Torii 2012, Han et al. 2022). One-day-old germinated seedlings were dissected from seeds and placed onto chamber slides (Thermo Fisher Scientific - Waltham, MA, USA, Nunc Lab-Tek II No. 155379), which were then placed on a motorized stage. Leica Stellaris 8 FALCON was used with the following conditions: CFP, excitation at 458 nm and emission from 464 to 510 nm; GFP, excitation at 488 nm and emission from 490 to 546 nm; YFP, excitation at 514 nm and emission from 520 to 560 nm and mCherry and tagRFP, excitation at 561 nm and emission from 570 to 620 nm. Signals were visualized sequentially using separate HyD detectors (HyDX/HyDS) in TauSeparation mode. Leica SP5 images were imaged using SP5-WLL/Argon with the following conditions: CFP, excitation at 458 nm and emission from 468 to 600 nm; GFP, excitation at 488 nm and emission from 490 to 546 nm; YFP, excitation at 514 nm and emission from 524 to 555 nm and mCherry, excitation at 560 nm and emission from 565 to 620 nm. For time-lapse imaging of isolated embryos, embryos were dissected from fully expanded mature green siliques and placed onto chamber slides, which were then placed on a motorized stage (Thermo Fisher Scientific, Nunc Lab-Tek II No. 155379) for long-term imaging. Leica Stellaris 8 FALCON was used with the following conditions: YFP excitation at 514 nm and emission from 520 to 560 nm and brightfield. Signals were visualized using HyD detectors (HyDX) in TauSeparation mode. For high-resolution imaging, still images obtained using either a 63× oil lens or a 20× dry lens with zoom factors of 1–4 have been used. The time-lapses for both germinating seedlings and mature embryos were collected at 30-min intervals using a 63× oil lens and zoom factors of 1.3–1.5, for high-resolution images and at 30-min intervals using a 40× oil lens or 20× dry lens and a zoom factor of 1 for data acquisition.

### Bioinformatic analysis and data visualization

For extracting the cell cycle genes from our MUTE ChIP-seq and RNA-seq data (Han et al. 2018, 2022), cell cycle–related genes were extracted from GO:0007049, GO:0051726, GO:0051321, GO:0007346, GO:0007050, GO:0000082, GO:0010389, GO:0010971, GO:0000086, GO:0045787, GO:0071158, GO:0000278, GO:0006267, GO:0007093, GO:0045786, GO:0045931, GO:0051446, GO:0007113, GO:0044843, GO:0045930, GO:0051445, GO:0060154, GO:0060184, GO:1900087, GO:1902749 and GO:0090266 and were combined with manually curated genes. Genes increased and/or decreased by MUTE more than log 2 FC (fold change) 0.4 and targeted by MUTE were extracted. The data obtained were visualized using R-package ‘Venn Diagram’. The bedGraph file from previous MUTE ChIP-seq was generated and visualized in the Integrated Genomic Viewer browser (ver. 2.4.11) (Robinson et al. 2011).

### Image processing and quantitative analysis

A series of either z-stack confocal images (time-lapse imaging) or single-plane images were obtained to capture fluorescent protein signals (CFP, GFP, YFP and mCherry). Raw data were collected with 512 × 512-pixel images and imported into Fiji-ImageJ v1.8.0_66 to generate RGB images/z-stacks using the channel merge function. To correct the drift of multichannel z-stacks, the ‘StackReg’ plugin was applied. Movies are played with 7 fps. The area of meristemoids was quantified using Fiji-ImageJ v2.3. Statistical analyses were performed using GraphPad Prism v9.2. For two-sample comparisons, Student’s *t*-tests were performed. Graphs were generated using GraphPad Prism v9.2. The value of *n*, the number of each experiment or sample and how statistical significance was defined are indicated in each relevant figure legend. The exact values and correlation statistics are listed in **Supplementary Dataset S2**.

## Supplementary Material

pcad002_Supp

## Data Availability

The data underlying this article (original iMUTE RNA-seq and MUTE ChIP-seq datasets) are available in NCBI GEO (National Center for Biotechnology Information Gene Expression Omnibus) at https://www.ncbi.nlm.nih.gov/geo/query/acc.cgi?acc=GSE107018 and https://www.ncbi.nlm.nih.gov/geo/query/acc.cgi?acc=GSE173338, and they can be accessed with GSE107018 and GSE173338, respectively.
