# Supplementary material for: Cell Cycle Dynamics during Stomatal Development: Window of MUTE Action and Ramification of Its Loss-of-Function on an Uncommitted Precursor
Source: Plant Cell Physiol. 2023 Jan 4;64(3):325–35. doi: 10.1093/pcp/pcad002 (PMC10016323; doi:10.1093/pcp/pcad002)
Supplement: pcad002_Supp [file pcad002_supp.zip › suppl_data/pcp-2022-e-00250-File023.pdf]

Cell cycle dynamics during stomatal development: Window of MUTE action and ramification of its loss-of-function on an uncommitted precursor

Daniel T. Zuch[1†], Arvid Herrmann[1†], Eun-Deok Kim[1,2], Keiko U. Torii[1,2*]

Supplementary Materials Table of Contents

Supplementary Figures S1-S8 and Legends

Supplementary Videos S1-S6 and Legends

Supplementary Dataset S1-S2 and Title

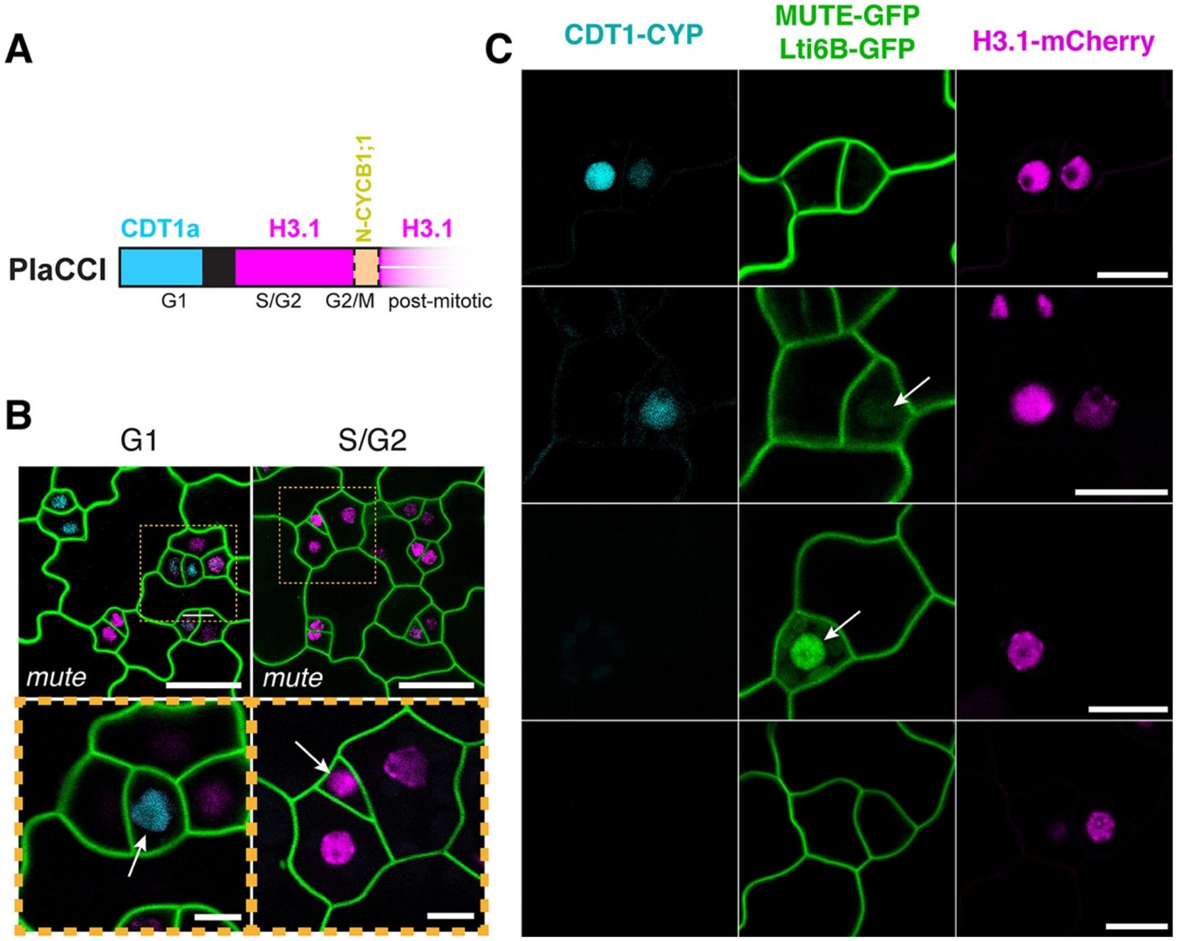

Supplementary Fig. S1: Higher resolution confocal images showing the expression of multiple markers in the stomatal precursor cells

(A) PlaCCI color code (adapted from Han et al.2022): Cyan: CDT1a-CFP, onset of G1 phase; Black: short period with no fluorescent signal; magenta: H3.1/HTR13-mCherry, S/G2 through late M, orange: CYCB1;1-YFP. Postmitotic refers to G1 or G0 (terminal division).

(B) Representative confocal microscopy images of abaxial epidermis from 3-4 day-old cotyledon of *mute* mutant seedlings expressing the cell cycle marker PlaCCI (cyan=G1, magenta=S/G2/lateM) and cell membrane marker Lti6B-GFP (top). Orange dotted areas in the upper panel are enlarged in the bottom panel. White arrows point to nucleus expressing either CDT1a-CFP (left bottom) or H3.1-mCherry (right bottom). Scale bars = 20 μm (top), 5 μm (bottom).

(C) Single still images of 3-day-old old cotyledon of Col-0 wild-type expressing MUTE-GFP (green nucleus), cell cycle marker PlaCCI and cell membrane marker Lti6B-GFP. Note that MUTE-GFP expression turns on during G1 phase (CDT1a-CFP, arrow point to nucleus) and vanishes during S/G2 phase (H3.1-mCherry). Scale bars = 10 μm

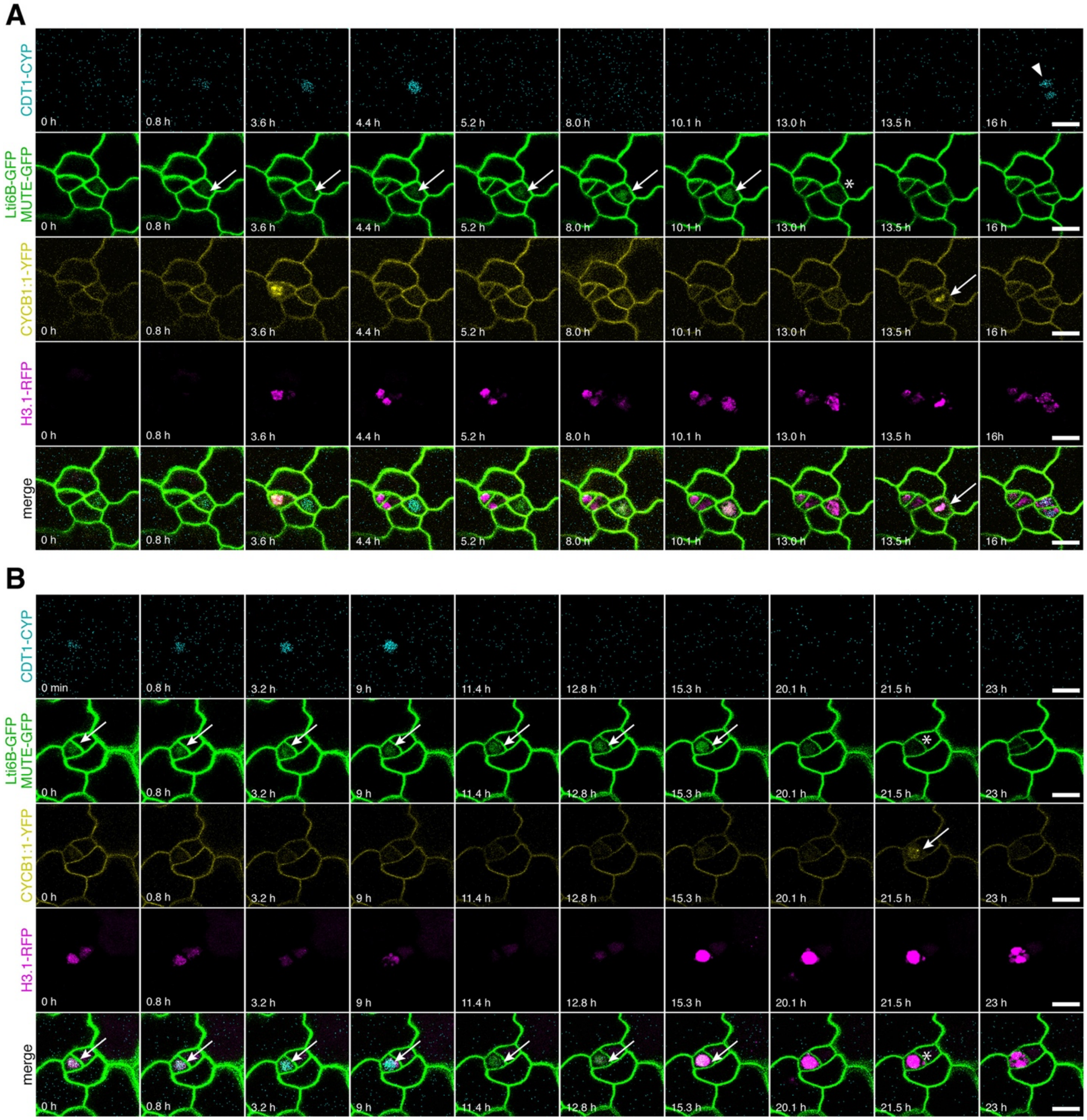

Supplementary Figure S2: MUTE-GFP and cell-cycle dynamics during terminal symmetric cell division of a GMC

Representative time-lapse images of WT cotyledons expressing the cell cycle marker PlaCCI, MUTE-GFP (green, nucleus) and Lti6B-GFP (green outline, plasma membrane) (A, B). Related to Fig. 2, Supplementary Videos S1, S2. MUTE-GFP accumulates at the onset of G1 (CDT1a-CFP, co-localization) within the nucleus (arrow points to nucleus) and degrades (asterisk) before the appearance of CYCB1;1-YFP (arrow, YFP channel). Scale bars = 10 μm

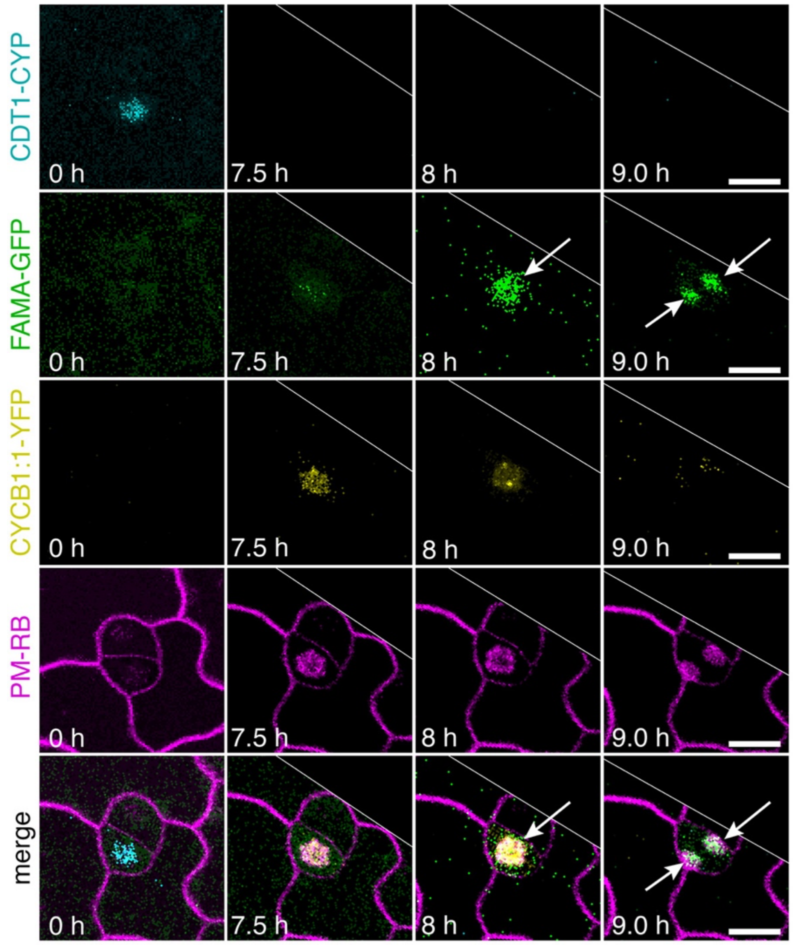

Supplementary Figure S3: FAMA-GFP and cell-cycle dynamics during terminal symmetric cell division of a GMC

Representative time-lapse images of WT plants expressing the cell cycle marker PlaCCI, FAMA-GFP (green, nucleus) and PM-RB (magenta outline). Note that FAMA-GFP starts to accumulate during S/G2 phase and colocalizes with CYCB1:1-YFP and H3.1-RFP. Scale bars = 10 μm

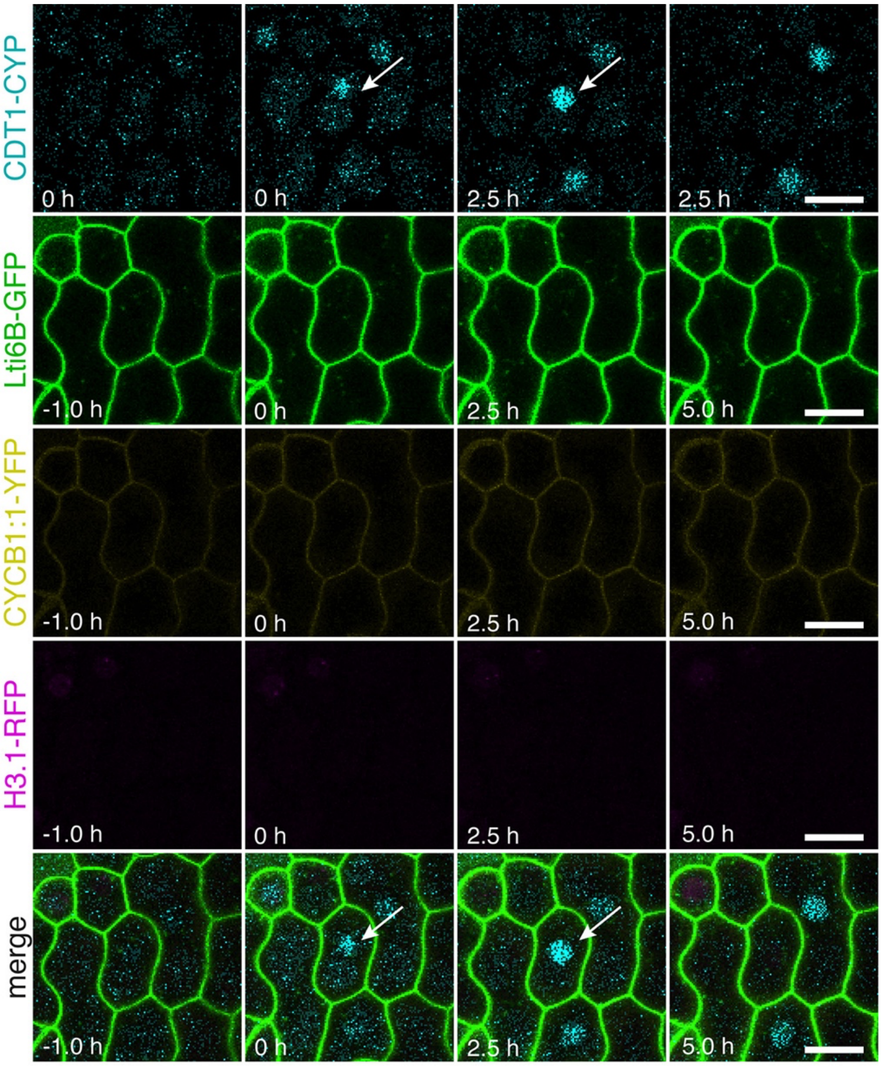

Supplementary Figure S4: G1 phase of a meristemoid undergoing ACD

Representative time-lapse images from 1-to 2dag abaxial cotyledons expressing both the cell cycle marker PlaCCI and Lti6B -GFP (green) in wild-type (Col-0) plants. Related to Fig. 5, Videos S5, S6. Arrow points to nucleus expressing CDT1a-CFP, indicative of a cell in G1 phase of the cell cycle. Scale bars = 10 μm

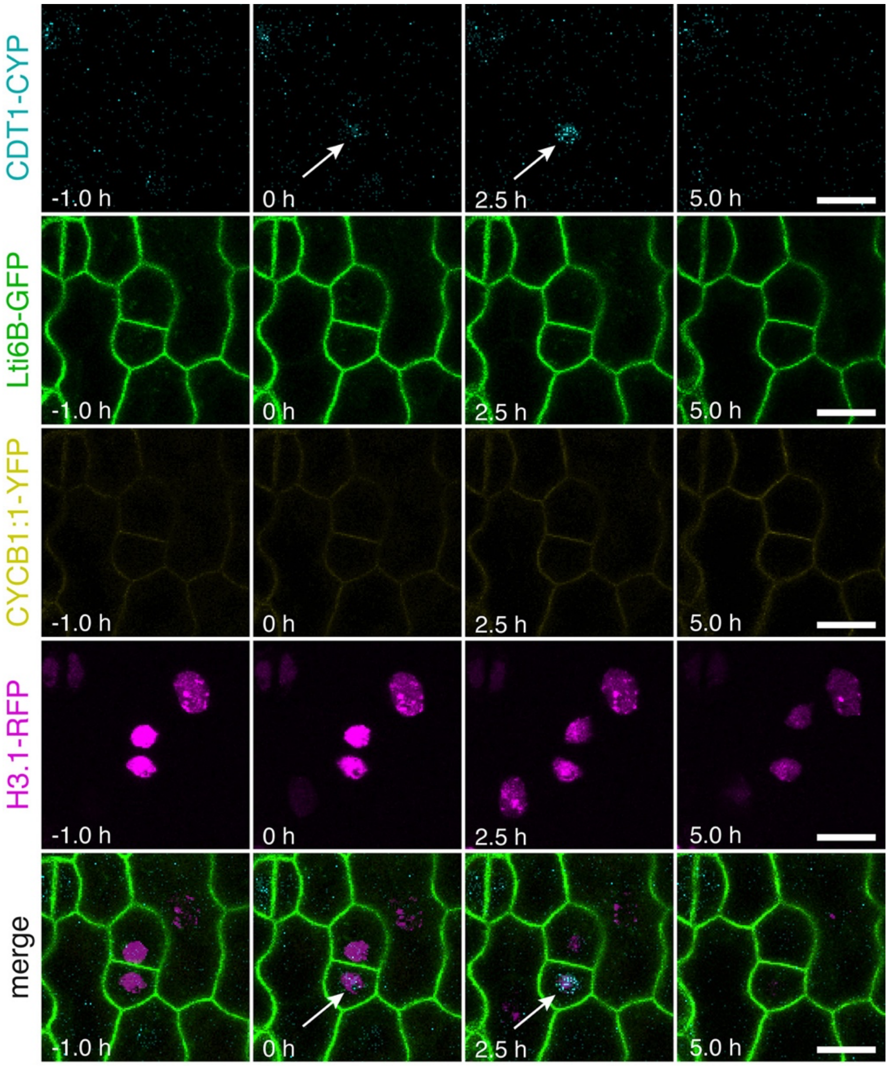

Supplementary Figure S5: G1 phase of a meristemoid undergoing successive ACD.
Daughter cell from ACD in Fig. S5. Representative time-lapse images from 2-to 3day-old abaxial cotyledons expressing both the cell cycle marker PlaCCI and Lti6B -GFP (green) in wild-type (Col-0) plants undergoing asymmetric cell division. Related to Fig. 4, Video S3, Video S5 and Video S6. Arrow points to nucleus expressing CDT1a-CFP, indicative of a cell in G1 phase of the cell cycle. Scale bars = 10 μm

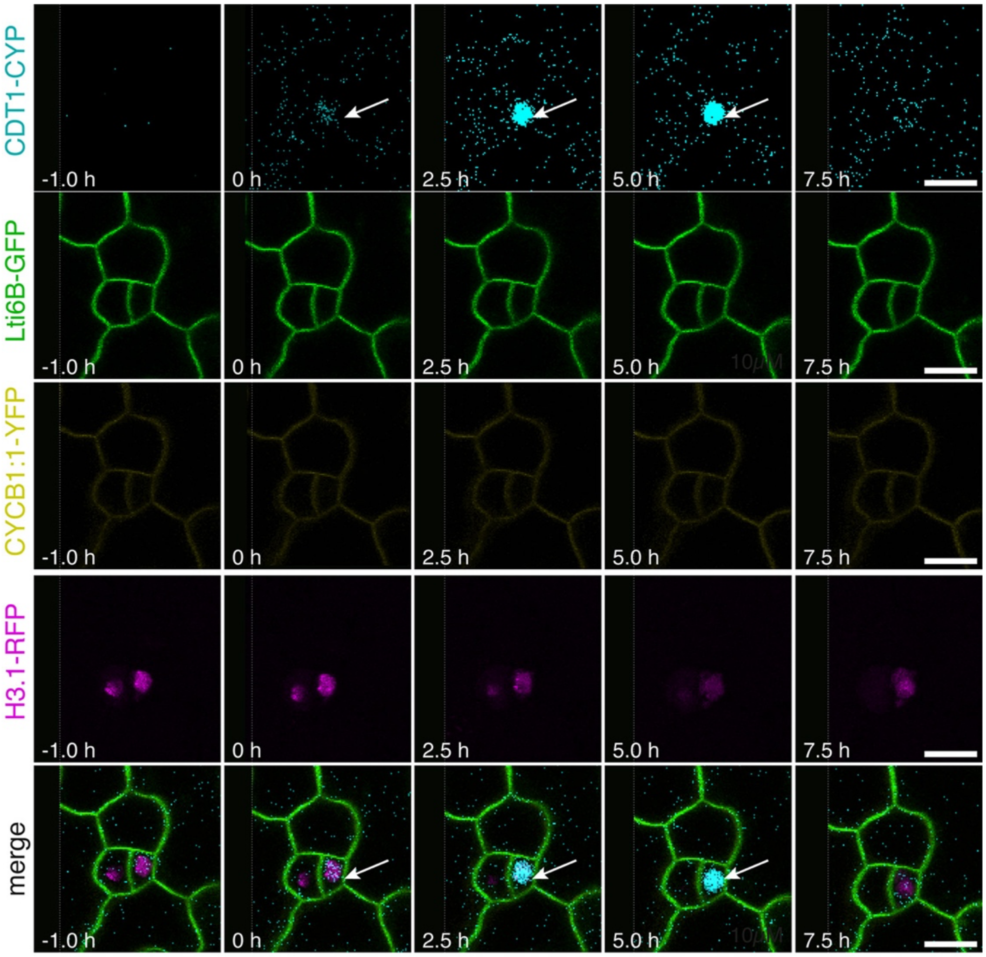

Supplementary Figure S6: G1 phase of a meristemoid undergoing terminal SCD. Daughter cell from ACD in Fig. S6. Representative time-lapse images from 2-to 3day-old abaxial cotyledons expressing both the cell cycle marker PlaCCI and Lti6B -GFP (green) in wild-type (Col-0) plants undergoing symmetric cell division. Related to Figure 4, Video S3, Video S5 and Video S6. Arrow points to nucleus expressing CDT1a-CFP, indicative of a cell in G1 phase of the cell cycle. Scale bars = 10 μm

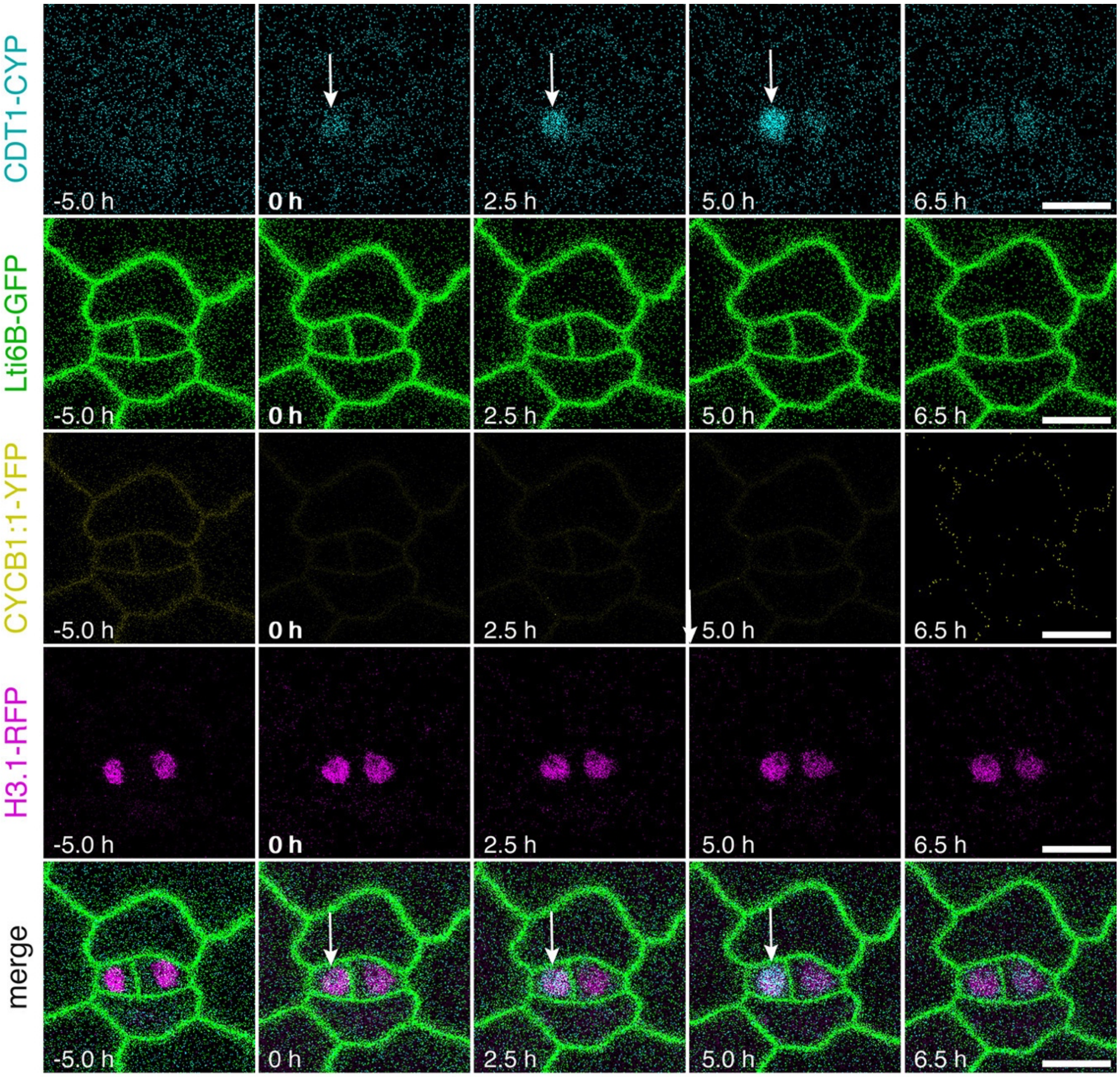

Supplementary Figure S7: G1 phase of a *mute* mutant meristemoid undergoing ACD. Representative time-lapse images from 1-to 5-day-old abaxial cotyledons expressing both the cell cycle marker PlaCCI and Lti6B -GFP (green) in *mute* mutants. Relate to Figure4, Video S3, Video S5 and Video S6. Meristemoid undergoing asymmetric cell division. Arrow points to a nucleus expressing CDT1a-CFP, indicative of a cell in G1 phase of the cell cycle. Scale bars = 5 μm

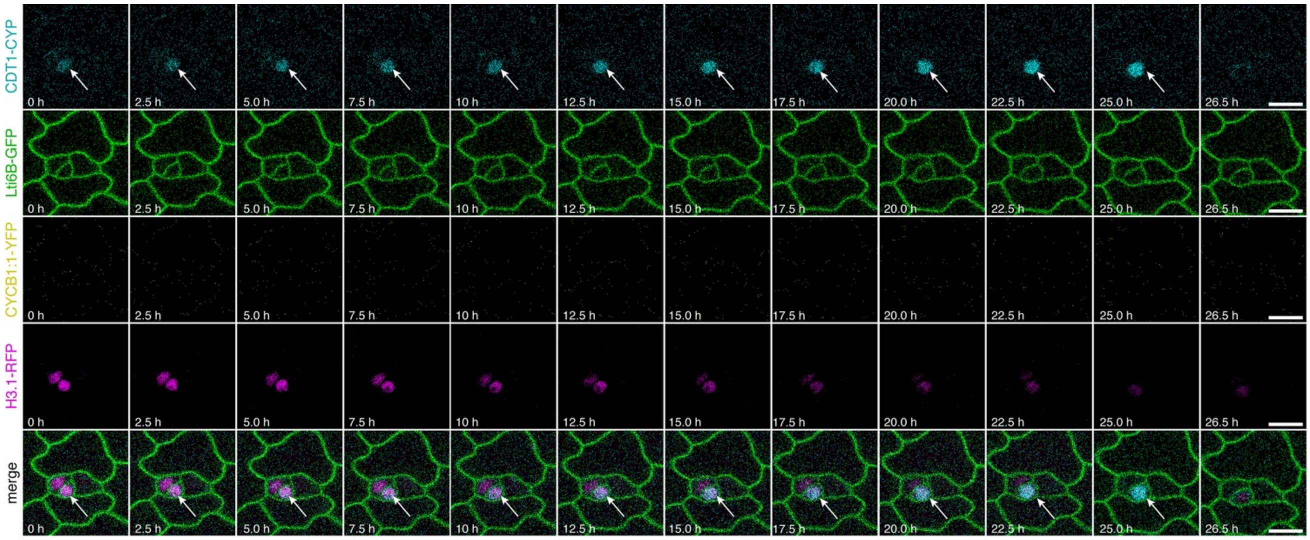

Supplementary Figure S8: G1 phase of a *mute* meristemoid undergoing successive ACDs. Daughter cell from ACD in Fig. S8. Representative time-lapse images from 1-to 5-day-old abaxial cotyledons expressing both the cell cycle marker PlaCCI and Lti6B -GFP (green) in *mute* mutants. Relate to Figure4, Video S3, Video S5 and Video S6. Note the extended G1 phase (CDT1a-CFP, arrow points to nucleus). Scale bars = 5 μm

Supplementary Video S1: MUTE-GFP and cell-cycle dynamics during terminal symmetric cell division of a GMC – Cell 1

Cell cycle marker PlaCCI and MUTE-GFP during a representative GMC undergoing terminal SCD in wild-type cotyledon epidermis. Corresponds to Supplementary Fig. S2A. Epidermal abaxial GMC directly undergoing terminal SCD after 2dag, expressing cell cycle marker PlaCCI (cyan = G1, magenta = S/G2/lateM), MUTE-GFP (green nucleus) and the membrane marker Lti6B-GFP. Small maximum projections for all channels of time-lapse recordings taken between 24 to 29 min time interval, 24h after preparation of an abaxial cotyledon epidermis. Played back at 7fps. Hours are indicated at the top in 24 to 29 min intervals. Scale bar = 5 μm

Supplementary Video S2: MUTE-GFP and cell-cycle dynamics during terminal symmetric cell division of a GMC – Cell 2

Cell cycle marker PlaCCI and MUTE-GFP during a representative GMC undergoing terminal SCD in wild-type cotyledon epidermis. Corresponds to Supplementary Fig. S2B. Epidermal abaxial GMC directly undergoing terminal SCD after 2 dag, expressing cell cycle marker PlaCCI (cyan = G1, magenta = S/G2/lateM), MUTE-GFP (green nucleus) and the membrane marker Lti6B-GFP (green). Small maximum projections for all channels of time laps recordings taken between 24 to 29 min time interval 24 h after preparation of an abaxial cotyledon epidermis. Played back at 7 fps. Hours are indicated at the top in 24 to 29 min intervals. Scale bar = 5 μm

Supplementary Video S3: Cell cycle dynamics of a pre-formed embryonic GMC during terminal SCD

Cell cycle marker PlaCCI during a representative pre-formed GMC undergoing terminal SCD in wild-type cotyledon epidermis. Related to Fig. 2. Epidermal abaxial GMC directly undergoing terminal SCD after 2 dag, expressing cell cycle marker PlaCCI (cyan = G1, magenta = S/G2/lateM) and the membrane marker Lti6B-GFP (green). Small maximum projections for all channels of time-lapse recordings taken at 30 min time interval 24 h after preparation of an abaxial cotyledon epidermis. Played back at 7 fps. Hours are indicated at the top in 30 min intervals. Scale bar = 5 μm

Supplementary Video S4: *MUTE* promoter activity in isolated embryo in culture

MUTEpro::nucYFP expression in cotyledon epidermis from an isolated Walking-Stick stage embryo in culture. The video sequence captures both a representative, pre-formed GMC undergoing terminal SCD (left) and a *de novo* ACD (right). Related to Fig. 2. Small maximum projections for YFP and brightfield channels of time-lapse recordings taken at 30 min time interval immediately after dissecting from fully expanding mature green siliques Played back at 7 fps. Hours are indicated at the top in 30 min intervals. Scale bar = 10 μm

Supplementary Video S5: Cell cycle dynamics during successive WT meristemoid ACDs and SCD

Cell cycle marker PlaCCI during representative, cumulative ACDs and terminal SCD in wild-type cotyledon epidermis. Related to Figure4. Two rounds of asymmetric cell divisions (ACDs) and one final terminal SCD in Col-0 expressing the cell cycle marker PlaCCI (cyan = G1, magenta = S/G2/lateM) and the membrane marker Lti6B-GFP (green). Small maximum projections for all channels of time-lapse recordings taken at 30 min time interval, 24h after preparation of an abaxial cotyledon epidermis. Played back at 7 fps. Hours are indicated at the top in 30 min intervals. Scale bar = 5 μm

Supplementary Video S6: Cell cycle dynamics during successive *mute* meristemoid ACDs

Cell cycle marker PlaCCI during representative, cumulative ACDs in *mute* cotyledon epidermis. Related to Figure4. Two rounds of asymmetric cell divisions (ACDs) in *mute* mutants expressing the cell cycle marker PlaCCI (cyan = G1, magenta = S/G2/lateM) and the membrane marker Lti6B-GFP (green). Small maximum projections for all channels of time-lapse recordings taken at 30min time intervals, 48 h after preparation of an abaxial cotyledon epidermis. Played back at 7 fps. Hours are indicated at the top in 30 min intervals. Scale bar = 5 μm

Supplementary Dataset S1: List of MUTE-bound, *iMUTE* up/down and Cell Cycle Genes

Supplementary Dataset S2: Quantitative Data and Statistics
